# Multi-ancestry genome-wide association meta-analysis of buprenorphine treatment response

**DOI:** 10.1038/s41386-025-02117-z

**Published:** 2025-05-06

**Authors:** Christal N. Davis, Yousef Khan, Richard C. Crist, Rachel Vickers-Smith, Emily E. Hartwell, Joel Gelernter, Kyle Kampman, Rachel L. Kember, Anne Le Moigne, Celine M. Laffont, Henry R. Kranzler

**Affiliations:** 1 Mental Illness Research, Education and Clinical Center, Veterans Integrated Service Network 4, Crescenz Veterans Affairs Medical Center, Philadelphia, PA 19104 USA; 2 Department of Psychiatry, University of Pennsylvania Perelman School of Medicine, Philadelphia, PA 19104 USA; 3 Department of Epidemiology, University of Kentucky College of Public Health and Center on Drug and Alcohol Research, Department of Behavioral Science, University of Kentucky College of Medicine, Lexington, KY 40536 USA; 4 Veterans Affairs Connecticut Healthcare System, West Haven, CT 06516 USA; 5 Department of Psychiatry, Yale University School of Medicine, New Haven, CT 06510 USA; 6 Indivior Inc., North Chesterfield, Chesterfield, VA 23235 USA

**Keywords:** Predictive markers, Psychiatric disorders

## Abstract

Although the mu-opioid partial agonist buprenorphine is increasingly being prescribed to treat opioid use disorder, patients’ responses to the drug vary and few clinical and no genetic predictors of treatment response have been identified. We conducted a genome-wide association study (GWAS) meta-analysis of buprenorphine treatment response (defined using urine drug screen results) in 4394 Veterans with opioid use disorder from the VA Million Veteran Program (751 of African-like ancestry [AFR] and 3643 of European-like ancestry [EUR]) and 296 participants from a clinical trial of extended-release buprenorphine (n_AFR_ = 104, n_EUR_ = 192). We conducted within-ancestry GWAS in both cohorts, followed by cross-ancestry, fixed-effects GWAS meta-analyses within and across cohorts. We also examined associations between demographic and clinical characteristics and buprenorphine treatment response. The cross-ancestry meta-analysis of both cohorts identified one genome-wide significant locus (rs149319538*)* that maps to *SLC39A10*, a gene that encodes a zinc transporter. Phenome-wide association analyses of the lead variant implicated connectivity of the uncinate fasciculus, a limbic white matter fiber tract. Of the clinical characteristics, only the presence of chronic pain and a lower maximum buprenorphine dosage were related to higher odds of treatment response in adjusted models. We report here the first genome-wide significant variant associated with buprenorphine treatment response. Larger samples are needed to replicate these findings and identify additional clinical and genetic factors that predict buprenorphine treatment efficacy to enable the use of a precision approach to OUD treatment.

## Introduction

In 2022, more than six million U.S. individuals aged 12 or older were diagnosed with an opioid use disorder (OUD) [1]. Individuals who misuse opioids are at high risk of an opioid overdose, which in 2023 resulted in about 81,083 U.S. deaths [2]. Three medications are currently approved by the Food and Drug Administration (FDA) to treat OUD: the opioid agonists methadone and buprenorphine and the opioid antagonist naltrexone [3]. Both methadone, a full opioid agonist, and buprenorphine, a partial opioid agonist, alleviate withdrawal symptoms and craving by binding to opioid receptors in the brain without producing the intense high resulting from other opioids. Despite their pharmacological differences, both drugs reduce opioid use and overdose risk [4, 5]. While methadone can be prescribed for treating OUD only in federally sanctioned opioid treatment programs, buprenorphine can be prescribed by any credentialed clinician, leading to a more rapid increase in the prescribing rate of buprenorphine than of methadone [6].

The factors contributing to variation in buprenorphine treatment outcomes are complex, encompassing demographic, clinical, and genetic factors. Poor adherence to treatment with buprenorphine is a key factor that limits its efficacy, reducing the prevalence of long-term abstinence in buprenorphine-treated patients [7, 8]. Extended-release buprenorphine (BUP-XR) was developed to enhance adherence by providing sustained therapeutic concentrations of the drug over a one-month period. Clinical and demographic factors, such as a history of hepatitis C (HCV) infection, underdosing, and younger age, also predict a poorer response [9, 10]. Understanding the impact of these and other factors is critical for enhancing the effectiveness of buprenorphine therapy and tailoring treatment to the needs of individuals.

Most genetic research on the response to buprenorphine treatment has employed candidate-gene approaches, often in small samples. Several studies of variants in the delta-opioid receptor gene (*OPRD1*) showed associations with treatment response [11–14]. The largest of these included 566 individuals of European-like (EUR) ancestry and 77 individuals of African-like (AFR) ancestry [12]. The first study to adopt a hypothesis-free approach with a genome-wide association study (GWAS) of buprenorphine response [9], conducted in a sample of 1616 Veterans of EUR ancestry with OUD, identified no genome-wide significant (GWS) loci.

Using a larger sample to provide greater statistical power, we conducted a cross-ancestry GWAS meta-analysis combining data from a cohort of buprenorphine-treated Veterans with OUD (n_AFR_ = 751 and n_EUR_ = 3643) within the Million Veteran Program (MVP) and patients from a clinical trial of extended-release buprenorphine (n_AFR_ = 104 and n_EUR_ = 192) [15]. This meta-analysis represents the largest, and first multi-ancestry, GWAS of buprenorphine treatment response. These initial findings provide proof of concept of a more robust and comprehensive understanding of the genetic factors influencing therapeutic outcomes for OUD and pave the way for advancing a precision medicine approach to buprenorphine treatment.

## Materials and methods

This study complies with all relevant ethical regulations. It was approved by both the VA Central Institutional Review Board (IRB) and IRBs at all MVP sites. For the Indivior cohort, a centralized IRB reviewed and approved the study protocol. All patients provided written informed consent to participate.

### MVP cohort

The MVP is a large biobank created and maintained by the U.S. Department of Veterans Affairs (VA). The latest release of MVP data includes electronic health record (EHR) information on 963,753 Veterans, about two-thirds of whom also have available genotype data. Using cohort definitions from a previous GWAS in a smaller portion of the MVP sample [9], EHR data were queried to identify Veterans who had received at least one inpatient or two outpatient International Classification of Diseases (ICD)-9/10 diagnosis codes for OUD and for whom there was genotype data and evidence of at least 28 consecutive days of buprenorphine exposure. Exposure was defined using evidence of filled prescriptions for buprenorphine. For individuals with more than one buprenorphine exposure period, we used data from only the first period. The exposure window for each patient started the day the buprenorphine prescription was filled and ended either when more than two weeks had passed with no new buprenorphine prescription, or a maximum of 180 days had passed. The buprenorphine treatment windows for this study occurred between August 4, 2003 and October 28, 2023.

**Fig. 1:**
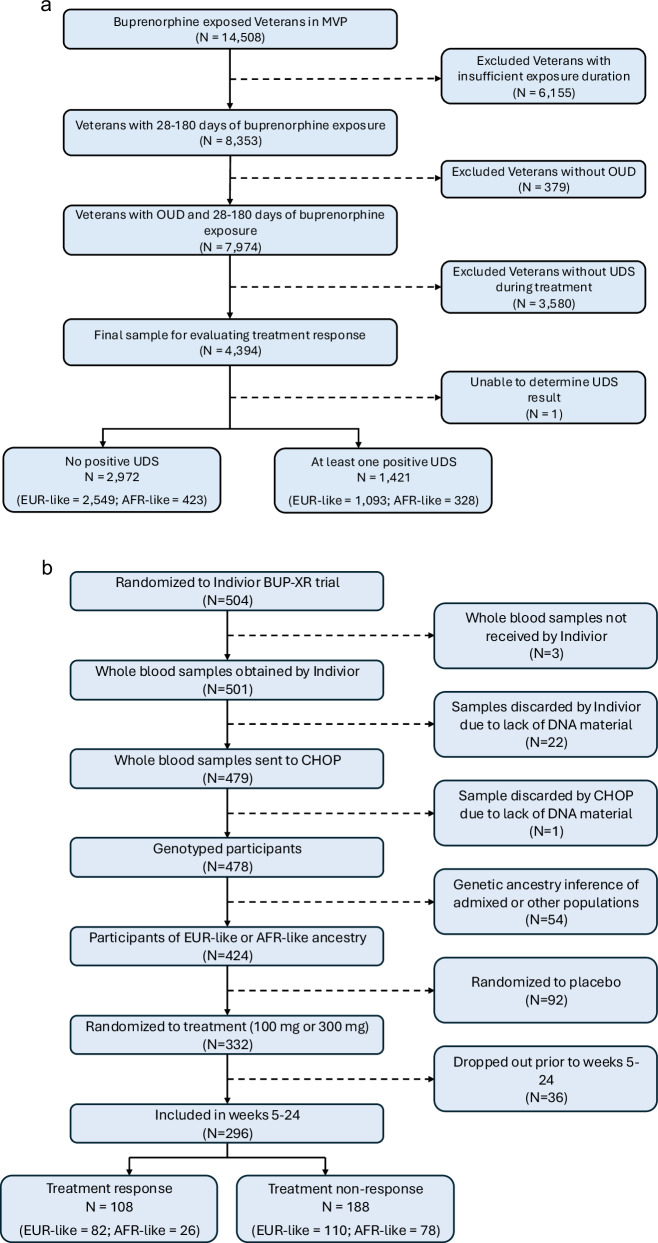
Flow diagram. Flowchart of inclusion/exclusion criteria for (**a**) the Million Veteran Program (MVP) and (**b**) Indivior cohorts. OUD opioid use disorder, UDS urine drug screen, CHOP Children’s Hospital ofPhiladelphia, EUR-like European-like ancestry, AFR-like African-like ancestry.

Urine drug screen (UDS) results included both qualitative and quantitative values that required reconciliation with cutoff thresholds. The median number of UDS completed during treatment was 16 (M = 23.85, SD = 24.40). Treatment responders were individuals with consistently negative UDS results for opioids (including methadone) throughout treatment. Despite variation in UDS reporting, outcome results for nearly all UDS records (~98%) were categorized and included in the analyses. We excluded one patient for whom there was no UDS obtained during treatment, which yielded a total of 4393 individuals (Fig. 1a).

### Indivior cohort

The Indivior cohort comprised participants in a randomized, double-blind placebo-controlled study of BUP-XR conducted in 36 U.S. treatment centers [15]. Participants were included if they were age 18–65 and had a Diagnostic and Statistical Manual of Mental Disorders, Fifth Edition (DSM-5) diagnosis [16] of moderate or severe OUD in the three months prior to study enrollment. Complete eligibility criteria have been described previously [15, 17].

Following a 3-day induction period, participants received at least 7 days of 8–24 mg daily of sublingual buprenorphine. If, during that time, they reported no significant opioid craving or withdrawal symptoms, they were randomly assigned to receive 6 monthly injections of 300 mg, 2 monthly injections of 300 mg plus 4 monthly injections of 100 mg, or volume-matched placebo doses for 6 months. Participants were seen weekly for 24 weeks. At each visit, a UDS, self-reported illicit drug use via the timeline follow-back method, measures of opioid withdrawal and craving, and safety assessments were obtained.

The primary efficacy endpoint in the clinical trial was the percentage of visits (through weeks 5–24) at which participants were opioid abstinent, defined as both UDS and self-reports of illicit opioid use being negative. The secondary endpoint was treatment success, a binary measure of the number of participants who achieved at least 80% opioid abstinence during weeks 5–24. For both endpoints, missing values during the period of weeks 5–24 were imputed as positive. Consistent with the clinical trial outcome definition, we defined treatment responders in the Indivior cohort as individuals who were abstinent for at least 80% of the time from weeks 5–24, with missing UDS values treated as positive. We excluded 27 individuals for whom DNA was not available and 54 individuals who were not of EUR or AFR ancestry, yielding a total of 296 participants for GWAS (Fig. 1b).

### Demographic and clinical association analyses

In the MVP cohort, we queried EHR data for comorbid disorders and correlates of treatment response using the same approach as in our previous GWAS [9]. We identified ICD-9/10 codes (Supplementary Methods) for the following comorbid disorders: human immunodeficiency virus (HIV), hepatitis C (HCV) infection, anxiety, depression, posttraumatic stress disorder (PTSD), and chronic pain. We included only diagnoses received prior to or during the buprenorphine treatment window. We also extracted demographic information, including age, sex, body mass index (BMI), and self-reported race and ethnicity, and clinical information, including the median and maximum daily buprenorphine dosage calculated from the dosage of medication, the number of doses, and the number of days’ supply prescribed.

In the Indivior cohort, we used several measures as described in the appendix to the phase 3 BUP-XR trial [15]. Specific quantitative variables such as the Visual Analog Scale (VAS) craving score and Clinical Opiate Withdrawal Scale (COWS) score were measured at baseline, defined as the last non-missing value prior to the first BUP-XR injection. Demographics and BMI were obtained at screening, defined as 15–30 days prior to the first injection. Presence of comorbidities (HCV, depression, anxiety, bipolar, and any psychiatric disorder) and tobacco, alcohol, and cocaine use were identified from the medical history obtained at screening.

To examine associations of the clinical and demographic factors with treatment response, we conducted logistic regressions in RStudio [18]. Initial bivariate unadjusted models were run first, and phenotypes that were associated with treatment response at *p* < 0.1 were included together in an adjusted model. Analyses were run separately within each cohort.

### Genotyping, imputation, and GWAS

Procedures for genotyping and imputation in MVP participants have been described previously [19]. Briefly, genotype data were generated using a custom ThermoFisher Axiom MVP 1.0 platform. Imputation was performed using a hybrid reference panel of the African Genome Resources [20] and the 1000 Genomes Project [21]. Genetically inferred ancestry (GIA) was estimated using a random forest classifier trained on 1000 Genomes data and applied to the MVP principal component analysis (PCA) data [22].

Genotyping of Indivior participants was performed using the Illumina Infinium Global Screening Array-24 kit at the Center for Applied Genomics, Children’s Hospital of Philadelphia. The quality of genetic data was assessed by comparing genetically determined sex with self-reported sex, and identity-by-descent was analyzed to ensure unrelatedness of individuals (Supplementary Fig. 1). Imputation was performed on the TOPMed Imputation Server [23] using the 1000 Genomes Phase 3 reference panel [21]. PCA was performed on a merged dataset of Indivior and 1000 Genomes individuals, and GIA was assigned to Indivior participants based on the shortest Euclidean distance to the nearest center of each of five superpopulation groups (Supplementary Fig. 2). Only individuals of AFR or EUR ancestry were retained due to small numbers of individuals in other GIA groups.

GWAS were conducted within GIA groups in PLINK v2.0 [24] using logistic regression models. Genetic variants with a minor allele frequency >1%, missing call rates <5%, Hardy-Weinberg equilibrium >1 × 10^–6^, and imputation quality >0.7 were included. We used the first 10 within-ancestry PCs, age, and sex as covariates. In MVP, a sensitivity analysis included maximum daily buprenorphine dosage as an additional covariate due to the large variability in dosage among participants. In the Indivior sample, we performed a sensitivity analysis with treatment responders defined as those with consistently negative UDS throughout the treatment period to mirror the phenotyping procedures of the MVP sample (Supplementary Methods).

### GWAS meta-analysis

Cross-ancestry (AFR + EUR) meta-analyses were performed in each cohort separately using METAL [25]. For the cross-cohort meta-analysis, the within-ancestry GWAS results from the MVP and Indivior samples were first meta-analyzed together using fixed effects inverse-variance weighted meta-analysis. Next, cross-ancestry meta-analyses were performed on these results across the two samples. As a sensitivity analysis, we meta-analyzed results from the GWAS in the MVP cohort with the sensitivity analysis GWAS conducted in the Indivior sample (i.e., using a 100% abstinence threshold to define treatment response; Supplementary Methods). Genomic control corrections were applied when there was evidence of inflation (λ > 1). We performed linkage disequilibrium clumping of the cross-ancestry meta-analysis results using the 1000 Genomes phase 3 ALL reference panel with a window distance of 3000 kb and r^2^ threshold of 0.10.

### Post-GWAS analyses

Finemapping of the cross-ancestry meta-analysis was performed using the Bayesian approach standard in LocusZoom [26]. This method identifies the most likely causal variant using Bayes factors and assumes a single causal variant per locus. Using Functional Mapping and Annotation of GWAS (FUMA) [27], we performed gene mapping based on: (1) position (10 kb), (2) eQTLs (BrainSeq, PsychENCODE, BRAINEAC, and GTEx v8 tissue types), and (3) 3D chromatin interactions (HiC data from the adult cortex, dorsolateral prefrontal cortex, hippocampus, ventricles, and neural progenitor cells). We also calculated the observed scale single-nucleotide polymorphism (SNP)-based heritability using linkage disequilibrium score regression (LDSC). For lead SNPs identified in the cross-ancestry meta-analysis of MVP and Indivior, a SNP-based phenome-wide association study (PheWAS) was conducted using the *ieugwasr* package (https://github.com/MRCIEU/ieugwasr). To capture phenotypic associations of genetic liability more broadly, we also calculated polygenic scores (PGS) for buprenorphine treatment response in the Yale-Penn cohort using PRS-CS [28]. Given the GWAS sample’s limited size, we set the global shrinkage parameter to 0.01 as recommended by the software’s creators [28]. For other options, we used the default settings. A PheWAS was conducted separately for AFR and EUR individuals in the Yale-Penn sample using the within-ancestry meta-analysis results and procedures described previously [29]. A false discovery rate (FDR) correction was applied to the PheWAS results to account for multiple testing.

## Results

### Demographic and clinical factors

For both the MVP and Indivior samples, the only demographic factors associated with buprenorphine treatment response were EUR ancestry and self-reported White race, the effects of which were nonsignificant after adjusting for other characteristics (Tables 1, 2). In the MVP sample, a higher daily maximum buprenorphine dosage was associated with reduced odds of responding to treatment, which persisted in models adjusted for genetic ancestry, self-reported race, HCV, anxiety, depression, PTSD, and chronic pain (aOR [95% confidence interval (CI)] = 0.99 [0.98–0.995], *p* = 0.003). In unadjusted models, the presence of several comorbidities was related to treatment response, but only chronic pain remained significant following adjustment (aOR [95% CI] = 1.16 [1.01–1.33], *p* = 0.03), reflecting increased odds of buprenorphine treatment response among individuals with chronic pain. In the adjusted model for the Indivior sample, there were no significant associations with treatment response. Comorbid substance use and substance use disorders were not related to treatment response in the unadjusted or adjusted models in either cohort.

**Table 1:** Million Veteran Program sample characteristics and their associations with treatment response (*N* = 4394).

Variable	% or Mean	SD or N	Unadj. OR (95% CI)	*P* value	Adj. OR (95% CI)	*P* value
Age	49.62	13.43	0.99 (0.99–1.00)	0.44	–	–
Sex (ref = Male)			0.84 (0.68–1.05)	0.12	–	–
Female	8.81%	387				
Male	91.19%	4006				
Ancestry (ref = AFR)		**1.81 (1.54 - 2.12)**	**4.95** **×** **10**^**–13**^	1.13 (0.65–1.96)	0.67	
EUR	82.91%	3643				
AFR	17.09%	751				
Hispanic or Latine	1.08%	47	0.85 (0.47–1.57)	0.59	–	–
Self-reported race (ref = non-White)		**1.83 (1.56 - 2.14)**	**1.02** **×** **10**^–**13**^	1.64 (0.95–2.80)	0.07	
Black/African	16.75%	723				
American						
White	82.09%	3544				
Other	1.16%	50				
Treatment response	67.65%	2972	–	–	–	–
Median daily dosage (mg)	12.63	6.86	0.99 (0.99–1.00)	0.23	–	–
Max daily dosage (mg)	15.19	7.48	**0.99 (0.98–1.00)**	**0.01**	**0.99 (0.98–1.00)**	**<0.01**
Body mass index	28.66	5.53	1.01 (1.00–1.02)	0.19		
HIV	2.25%	99	1.05 (0.69–1.63)	0.82	–	–
Hepatitis C	31.13%	1368	**0.85 (0.74–0.97)**	**0.02**	0.94 (0.81–1.08)	0.38
Anxiety	38.39%	1709	**1.23 (1.08–1.40)**	**<0.01**	1.06 (0.92–1.23)	0.40
Depression	58.92%	2589	**1.24 (1.09–1.41)**	**<0.01**	1.15 (1.00–1.33)	0.06
Posttraumatic stress disorder	50.09%	2201	**1.18 (1.04–1.33)**	**0.01**	1.11 (0.97–1.27)	0.13
Other substance use disorder	63.25%	2779	0.91 (0.80–1.04)	0.18		
Chronic pain	49.54%	2177	**1.26 (1.11–1.43)**	**3.17** **×** **10**^**–4**^	**1.16 (1.01–1.33)**	**0.03**

Variables with a *p* value < 0.1 in unadjusted models were included in adjusted models. Bold indicates significance at *p* < 0.05.

*SD* standard deviation, *OR* odds ratio, *CI* confidence interval, *AFR* African-like genetically inferred ancestry, *EUR* European-like genetically inferred ancestry.

**Table 2:** Indivior sample characteristics and their associations with treatment response (*N* = 296).

Variable	% or Mean	SD or N	Unadj. OR (95% CI)	*P* value	Adj. OR (95%CI)	*P* value
Age	40.4	11.4	0.99 (0.97–1.01)	0.41	–	–
Sex (ref = Male)			1.10 (0.67–1.79)	0.72	–	–
Female	34.8%	103				
Male	65.2%	193				
Ancestry (ref = AFR)			**2.24 (1.33–3.84)**	**2.8 × 10**^**–3**^	0.90 (0.04–10.6)	0.93
EUR	64.9%	192				
AFR	35.1%	104				
Hispanic or Latine	2.02%	6	0.87 (0.12–4.52)	0.87	–	–
Self-reported race (ref = non-White)			**2.29 (1.36–3.92)**	**2.1 × 10**^**–3**^	2.54 (0.22–59.8)	0.47
Black/African	34.5%	102				
American						
White	64.5%	191				
Other	1.01%	3				
Treatment response	36.5%	108	–	–	–	–
Dose (ref = 100 mg)			1.0 (0.62–1.61)	1.00	–	–
100 mg	50.0%	148				
300 mg	50.0%	148				
Body mass index	25.8	4.45	1.04 (0.99–1.10)	0.15	–	–
Hepatitis C	14.2%	42	0.96 (0.48–1.88)	0.91	–	–
Depression	12.8%	38	1.02 (0.49–2.04)	0.96	–	–
Anxiety	10.8%	32	0.55 (0.22–1.22)	0.16	–	–
Bipolar disorder	2.03%	6	1.76 (0.32–9.67)	0.49	–	–
Any psychiatric disorder	19.9%	59	0.72 (0.38–1.31)	0.29	–	–
Current tobacco use	85.8%	254	0.58 (0.30–1.13)	0.11	–	–
Current alcohol use	54.7%	162	1.05 (0.66–1.70)	0.83	–	–
Current cocaine use	37.5%	111	0.91 (0.56–1.48)	0.71	–	–
Baseline VAS	6.35	12.6	0.99 (0.96–1.01)	0.27	–	–
Baseline COWS	2.33	2.90	0.94 (0.86–1.02)	0.18	–	–

Variables with a *p* value < 0.1 in unadjusted models were included in adjusted models. Baseline refers to the last score following sublingual buprenorphine induction but prior to the first buprenorphine extended-release injection. Bold indicates significance at *p* < 0.05.

*SD* standard deviation, *OR* odds ratio, *CI* confidence interval, *AFR* African-like genetically inferred ancestry, *EUR* European-like genetically inferred ancestry, *VAS* visual analog scale score at baseline, *COWS* clinical opiate withdrawal scale score.

### GWAS

**Fig. 2:**
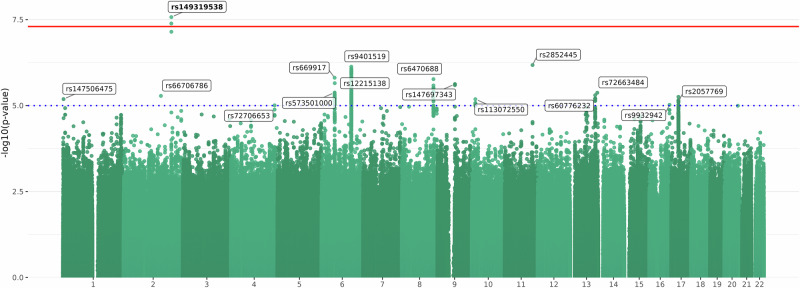
Manhattan plot of the cross-ancestry GWAS meta-analysis of the Million Veteran Program and Indivior samples. The blue dotted line indicates *p* < 1 × 10^–5^, and the solid red line represents the genome-wide significance threshold of *p* < 5 × 10^–8^. The lead variants for nominally and genome-wide significant loci are annotated, with the significant lead variant annotated in bold.

The primary GWAS, a cross-ancestry meta-analysis of both the MVP and Indivior cohorts, yielded two GWS SNPs—rs149319538 (lead SNP) and rs113613122—within a locus on chromosome 2 (Supplementary Fig. 3). Fine mapping indicated that both SNPs were within the credible set of causal variants for the locus (posterior probabilities of 0.491 and 0.329, respectively). Using FUMA, the GWS lead SNP was mapped to *SLC39A10*, a zinc transporter gene, based on 3D chromatin interaction data from neural progenitor cells (FDR *p* value = 4.64 × 10^–7^) and left cerebral ventricular cells (FDR *p* value = 1.04 × 10^–8^). Additionally, there were suggestive associations for 223 SNPs (*p* < 1 × 10^–5^), corresponding to 15 independent loci (Table 3 and Fig. 2). The meta-analysis had an observed scale SNP-based heritability of 0.18 (SE = 0.09, 95% CI = 0.02–0.35).

**Table 3:** Significant and suggestive lead SNPs from the cross-ancestry GWAS meta-analysis of the Million Veteran Program and Indivior cohorts.

Chromosome	Position	SNP	*P* value	Nearest Gene
2	196279963	rs149319538	2.66 × 10^–08^	*AC064834.1*
11	114180814	rs2852445	6.60 × 10^–07^	*NNMT*
6	122683440	rs9401519	7.36 × 10^–07^	*RNU1-18P*
6	54294688	rs669917	1.55 × 10^–06^	*CLNS1AP1*
8	130043829	rs6470688	1.70 × 10^–06^	*LINC00977*
6	54695917	rs12215138	2.22 × 10^–06^	*RP11-524H19.2*
9	71905345	rs147697343	2.37 × 10^–06^	*FAM189A2*
6	54365478	rs573501000	4.16 × 10^–06^	*CLNS1AP1*
13	111459167	rs72663484	4.23 × 10^–06^	*LINC00567*
13	102667059	rs60776232	5.00 × 10^–06^	*FGF14*
2	153461929	rs66706786	5.21 × 10^–06^	*FMNL2*
1	2563901	rs147506475	6.47 × 10^–06^	*MMEL1*
10	14767985	rs113072550	6.50 × 10^–06^	*FAM107B*
17	29691368	rs2057769	7.72 × 10^–06^	*NF1*
16	83173960	rs9932942	9.50 × 10^–06^	*CDH13*
4	179359799	rs72706653	9.81 × 10^–06^	*RNA5SP173*

The genome-wide significant SNP is bolded.

*SNP* single nucleotide polymorphism.

The MVP GWAS in AFR participants yielded no GWS loci (Supplementary Table 1 and Supplementary Fig. 4). The MVP GWAS in EUR participants yielded one GWS locus on chromosome 2, with the lead SNP identified as rs149319538 (*p* = 2.19 × 10^–8^). In a sensitivity analysis that included maximum daily buprenorphine dosage as a covariate, the lead SNP in EUR individuals remained significant, with no significant findings in the AFR GWAS (Supplementary Fig. 5). The cross-ancestry meta-analysis in the MVP cohort identified the same lead SNP—rs149319538 (*p* = 2.74 × 10^–8^)—as in the EUR GWAS (Supplementary Fig. 6). Within the Indivior sample, there were no GWS loci in either GIA group (Supplementary Fig. 7) or in the cross-ancestry GWAS meta-analysis (Supplementary Table 1 and Supplementary Fig. 8).

Findings for the sensitivity analysis that defined treatment response using a 100% abstinent threshold in the Indivior cohort were consistent with those of the primary GWAS. Within the Indivior sample, using the 100% abstinence treatment response threshold, GWAS identified no significant loci in either GIA group (Supplementary Fig. 9) or in the cross-ancestry meta-analysis (Supplementary Fig. 10). The cross-ancestry meta-analysis of the Indivior sensitivity analysis and MVP cohort yielded the same GWS locus as the primary meta-analysis (Supplementary Fig. 11).

### PheWAS

A PheWAS of rs149319538 identified 22 significant associations after Bonferroni correction (Supplementary Fig. 12). These included cardiovascular outcomes (i.e., ischemic stroke and hypertensive heart disease), pain-related conditions (i.e., arthritis and chondropathies), and an inflammatory marker (tumor necrosis factor ligand superfamily). Notably, multiple brain image-derived phenotypes (IDPs) were associated with the SNP. Greater white matter integrity in the uncinate fasciculus, a tract connecting the ventral prefrontal cortex (vPFC) with the amygdala, emerged as a consistent finding. Although prior to FDR correction for the PGS-based PheWAS there were 26 significant associations in EUR and 39 in AFR individuals, none remained significant after FDR correction (Supplementary Figs. 13, 14).

## Discussion

Combining data from AFR and EUR ancestry individuals from the MVP and a large clinical trial of BUP-XR, we identified the first GWS SNP associated with buprenorphine treatment response. Although located in an intergenic region, the lead SNP mapped to *SLC39A10* based on chromatin interaction data from neural progenitor and left cerebral ventricular cells. *SLC39A10* encodes the ZIP10 zinc transporter, which transports zinc into the cytoplasm from the extracellular space in brain, liver, erythroid and kidney tissues [30]. Emerging evidence from patient populations suggests a role for zinc in the response to opioids. For example, studies have shown that zinc deficiencies are prevalent in patients treated with opioids and that zinc-deficient patients often require a higher dosage of opioids to manage post-surgical pain [31]. In a randomized controlled trial of zinc supplementation in 68 patients receiving methadone treatment for OUD, supplemental zinc reduced depression, anxiety, and stress, while also decreasing the likelihood of substance use and craving [32]. These findings, combined with our genetic evidence, suggest that variation linked to *SLC39A10* may influence buprenorphine efficacy by affecting zinc transport and availability.

The lead SNP showed several phenotypic associations using publicly available GWAS data. Specifically, a greater number of effect alleles, indicating an increased propensity for treatment response, was associated in the UKBiobank (https://www.nealelab.is/uk-biobank) and FinnGen samples [33] with pain-related conditions like chrondropathies (e.g., osteoarthritis or spinal disc herniation). This PheWAS finding aligns with the phenotypic results from the MVP sample, where individuals with chronic pain had significantly higher odds of responding to buprenorphine treatment. Additionally, several brain IDPs were related to the lead SNP, including greater white matter integrity in the uncinate fasciculus, a tract that connects the vPFC and amygdala. Despite mixed findings [34], reduced white matter integrity in the uncinate fasciculus has been associated with adolescent binge alcohol and cannabis use [35], cognitive deficits in individuals with temporal lobe epilepsy [36], and social-emotional abnormalities in frontotemporal dementia [37]. Greater white matter integrity in the fiber bundle, as indicated by greater complexity in fiber orientation, may support emotion regulation and decision-making, potentially contributing to a better response to buprenorphine treatment in individuals with OUD. Further research is needed to understand how variation in white matter microstructure may influence treatment response.

The PGS-based PheWAS in the Yale-Penn sample identified possible associations with various non-opioid substance use, psychiatric, and sociodemographic phenotypes, although none were significant after multiple testing correction. A better understanding of the mechanisms underlying these associations may require larger samples but could help to differentiate patient subgroups that differ in their likelihood of benefitting from buprenorphine treatment. Such risk stratification could enable more targeted and effective treatment strategies for OUD.

In addition to genetic factors, two clinical characteristics—maximum daily dosage and comorbid chronic pain—were associated with treatment response in the MVP cohort. Flexible prescribing in clinical settings may explain why in MVP a higher maximum buprenorphine dosage was associated with a poorer treatment response, which contrasts with other findings that a lower dosage of buprenorphine is associated with poorer treatment outcomes [10]. That is, prescribers may start buprenorphine at a low dosage with a plan to increase it over time only in individuals whose OUD requires it. This approach may initially underdose some patients, resulting in a greater likelihood of relapse. Because we used a binary measure of abstinence as our treatment outcome, any relapse would result in the individual being classified as not responding to treatment. Consistent with the PheWAS finding of an association with pain-related conditions, individuals with chronic pain diagnoses were more likely to remain abstinent during buprenorphine treatment. Although in a meta-analysis buprenorphine was more effective at treating chronic pain in patients without OUD than those with OUD [38], even a modest pain reduction could increase treatment adherence and efficacy in OUD patients, potentially explaining the higher rate of treatment response observed in individuals with chronic pain. Future research should explore whether optimized early buprenorphine dosing and integrated pain management can enhance outcomes for individuals with OUD being treated with buprenorphine.

### Limitations

Despite the strengths of this study, including a comparatively large, multi-ancestry sample, several limitations should be noted. The frequency and timing of UDS varied across participants and cohorts, potentially leading to some misclassification of treatment response. Additionally, the use of EHR data in the MVP cohort prevented us from assessing treatment retention as an outcome, and we were unable to evaluate reliably treatment adherence among participants. In addition to variation in the reporting of buprenorphine treatment retention, the lack of systematic methods for collecting and testing UDS in the MVP cohort necessitated that we utilize a binary outcome (response vs. non-response) rather than a continuous measure of treatment response. The MVP data spanned two decades (2003–2023), during which time both the availability of prescribed opioids and the nature of the illicit opioid supply have changed substantially. Because of the limited sample size, we could not evaluate cohort effects, which could influence the findings. Whereas the sample is predominantly male, we could not address possible sex differences in genetic predictors of treatment response, which will be an important area for future research. Finally, although our GWAS meta-analysis increased statistical power relative to our previous GWAS in veterans [9], the complex genetic architecture of buprenorphine response likely involves many loci of small effect, necessitating considerably larger samples to uncover them.

## Conclusions

In the largest, and first multi-ancestry, GWAS of buprenorphine treatment response, we identified a novel genomic locus that may influence therapeutic outcomes in individuals with OUD. The lead SNP at this locus on chromosome 2 maps to *SLC39A10*, which encodes a zinc transporter, and was associated with greater white matter integrity in the uncinate fasciculus, a fiber tract connecting brain regions involved in emotional regulation and decision-making. In addition to the genetic factors that influence buprenorphine treatment response, we found that OUD patients with chronic pain had greater odds of responding to buprenorphine, likely due to its dual role as both an analgesic and maintenance treatment for OUD. These findings underscore the importance of considering both genetic and clinical factors that shape individual responses to buprenorphine treatment. Further research is needed to elucidate the underlying mechanisms of these associations and inform the development of personalized treatment strategies for OUD.

## Supplementary information

Supplementary Materials

Data set 1

## Data Availability

The full summary-level association data for the MVP sample are available through dbGaP: https://www.ncbi.nlm.nih.gov/projects/gap/cgi-bin/study.cgi?study_id=phs001672.v1.p1 (accession number phs001672.v1.p1). Data from the meta-analysis of the MVP and Indivior samples are available from https://zenodo.org/records/15103475.
