# Supplementary material for: Multi-ancestry genome-wide association meta-analysis of buprenorphine treatment response
Source: Neuropsychopharmacology. 2025 May 6;50(9):1346–53. doi: 10.1038/s41386-025-02117-z (PMC12260092; doi:10.1038/s41386-025-02117-z)
Supplement: Supplementary file 1 — Supplementary Materials [file 41386_2025_2117_MOESM1_ESM.docx]

**Supplementary Materials**

Davis et al., Multi-Ancestry Genome-Wide Association Meta-Analysis of Buprenorphine Treatment Response

[Supplementary Methods 2](#_Toc194071122)

[**International Classification of Diseases (ICD)-9 and 10 codes used** 2](#_Toc194071123)

[**Sensitivity analysis for Indivior sample GWAS & meta-analysis** 2](#_Toc194071124)

[Supplementary Figures 3](#_Toc194071125)

[**Supplementary Figure 1. Identity-by-descent values in Indivior dataset.** 3](#_Toc194071126)

[**Supplementary Figure 2. PCA plot of genetic ancestry determinations in Indivior subjects.** 4](#_Toc194071127)

[**Supplementary Figure 3. Regional plot of the top locus from the cross-ancestry GWAS meta-analysis.** 5](#_Toc194071128)

[**Supplementary Figure 4. Manhattan plots of the Million Veteran Program genome-wide association studies.** 6](#_Toc194071129)

[**Supplementary Figure 5. Manhattan plots of the Million Veteran Program genome-wide association studies controlling for maximum daily dosage.** 7](#_Toc194071130)

[**Supplementary Figure 6. Manhattan plot of the cross-ancestry Million Veteran Program genome-wide association meta-analysis.** 8](#_Toc194071131)

[**Supplementary Figure 7. Manhattan plots of the Indivior genome-wide association studies.** 9](#_Toc194071132)

[**Supplementary Figure 8. Manhattan plot of the cross-ancestry Indivior genome-wide association meta-analysis.** 10](#_Toc194071133)

[**Supplementary Figure 9. Manhattan plots of the Indivior genome-wide association sensitivity analyses.** 11](#_Toc194071134)

[**Supplementary Figure 10. Manhattan plot of the cross-ancestry Indivior genome-wide association sensitivity meta-analysis.** 12](#_Toc194071135)

[**Supplementary Figure 11. Manhattan plot of the cross-ancestry, cross-cohort genome-wide association sensitivity meta-analysis.** 13](#_Toc194071136)

[**Supplementary Figure 12. PheWAS results for the lead single-nucleotide polymorphism.** 14](#_Toc194071137)

[**Supplementary Figure 13. PheWAS of PGS in Yale-Penn within EUR individuals.** 15](#_Toc194071138)

[**Supplementary Figure 14. PheWAS of PGS in Yale-Penn within AFR individuals.** 16](#_Toc194071139)

# **Supplementary Methods**

## **International Classification of Diseases (ICD)-9 and 10 codes used**

| **Condition** | **ICD-9 Codes** | **ICD-10 Codes** |
| --- | --- | --- |
| **Opioid Use Disorder** | 304.0, 304.7, 305.5 | F11.1, F11.2 |
| **Human Immunodeficiency Virus** | 042, V08 | B20, Z21 |
| **Hepatitis C Infection** | 070.41, 070.44, 070.51, 070.54, 070.70, 070.71, V02.62 | B17.10, B17.11, B18.2, B19.20, B19.21, Z22.52 |
| **Anxiety** | 300, 300.01, 300.02, 300.09, 799.2 | F41, F41.0, F41.1, F41.8, F41.9 |
| **Depression** | 296.2, 296.3 | F32, F33 |
| **Post-Traumatic Stress Disorder** | 309.81 | F43.1 |
| **Substance Use Disorder (other than OUD)** | 303.0, 303.9, 305.1, 304.2, 304.3, 304.4, 304.1, 304.5, 304.6, 304.7, 304.8, 304.9 | F10.2, F17.2, F14.2, F12.2, F15.2, F13.2, F16.2, F18.2, F19.2 |
| **Chronic Pain** | 304.9, 307.89, 338.2, 338.4 | F45.42, G89.0, G89.2, G89.21, G89.22, G89.28, G89.29, G89.4, G90.5, M79.7 |

## **Sensitivity analysis for Indivior sample GWAS & meta-analysis**

The Million Veteran Program (MVP) dataset consists of observed clinical data recorded in an electronic medical record whereas the Indivior sample reflects a strictly controlled randomized clinical trial. For this reason, and to maintain consistency with the outcome reported for the Indivior clinical trial [1], we used different phenotyping procedures in the Indivior and MVP samples for optimal categorization of treatment response status. For the primary analysis, phenotyping was performed as described in the manuscript. We then conducted sensitivity analysis using similar definitions of treatment response in the MVP and Indivior samples: While we maintained the same definition of treatment response in the MVP sample, we redefined treatment response in the Indivior sample to include only individuals with consistently negative Urine Drug Screen results for opioids throughout the treatment period. We performed GWAS in the Indivior sample using this definition, which included 332 participants with a treatment response rate of 27.7% (n = 92). We then meta-analyzed this result with those from GWAS of the MVP sample to compare the results between our main analysis and sensitivity analysis.

**Reference**

1 Haight BR, Learned SM, Laffont CM, Fudala PJ, Zhao Y, Garofalo AS, et al. Efficacy and safety of a monthly buprenorphine depot injection for opioid use disorder: a multicentre, randomised, double-blind, placebo-controlled, phase 3 trial. Lancet. 2019;393(10173):778-90.

# **Supplementary Figures**


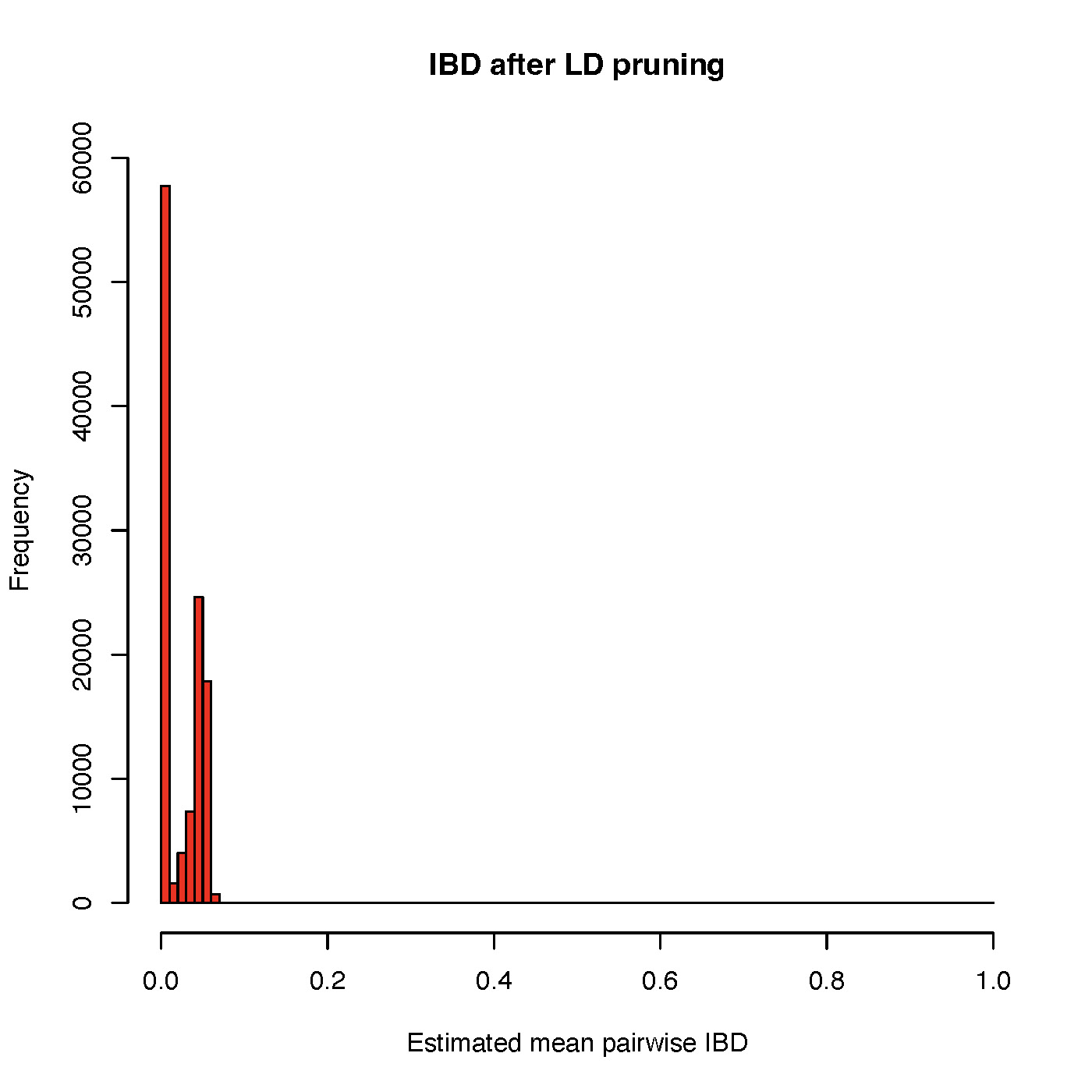


## **Supplementary Figure 1. Identity-by-descent values in Indivior dataset.**

Identity-by-descent was estimated for each pair of individuals included in GWAS, with the y-axis depicting the number of pairs with the corresponding identity-by-descent value denoted on the x-axis.**
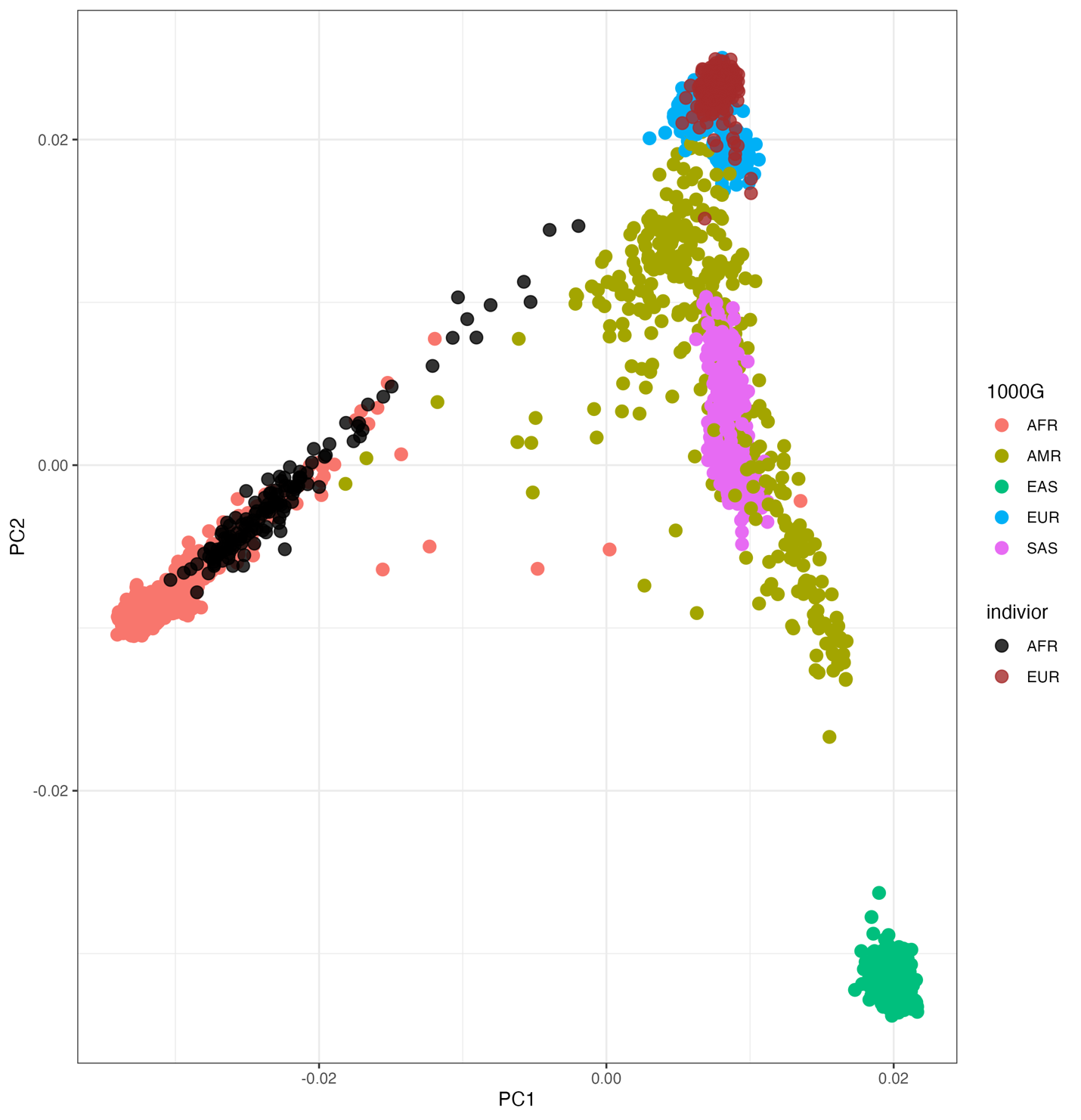
**

## **Supplementary Figure 2. PCA plot of genetic ancestry determinations in Indivior subjects.** PCA was performed on the merged 1000 genomes and Indivior dataset, with the x-axis representing the first principal component, and y-axis representing the second principal component.


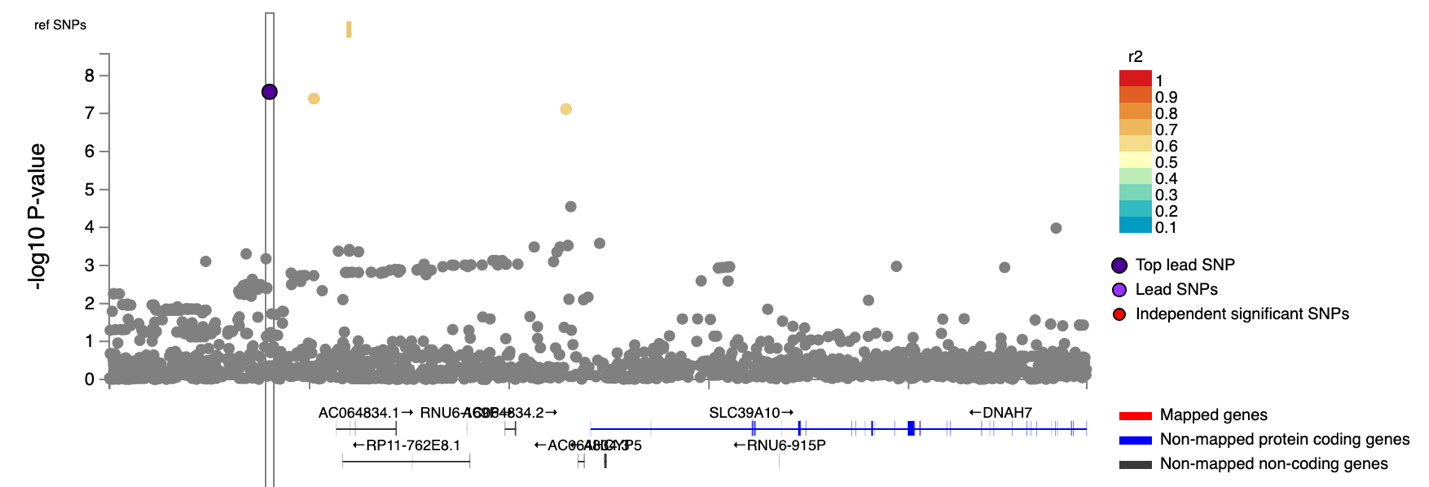


## **Supplementary Figure 3. Regional plot of the top locus from the cross-ancestry GWAS meta-analysis.**


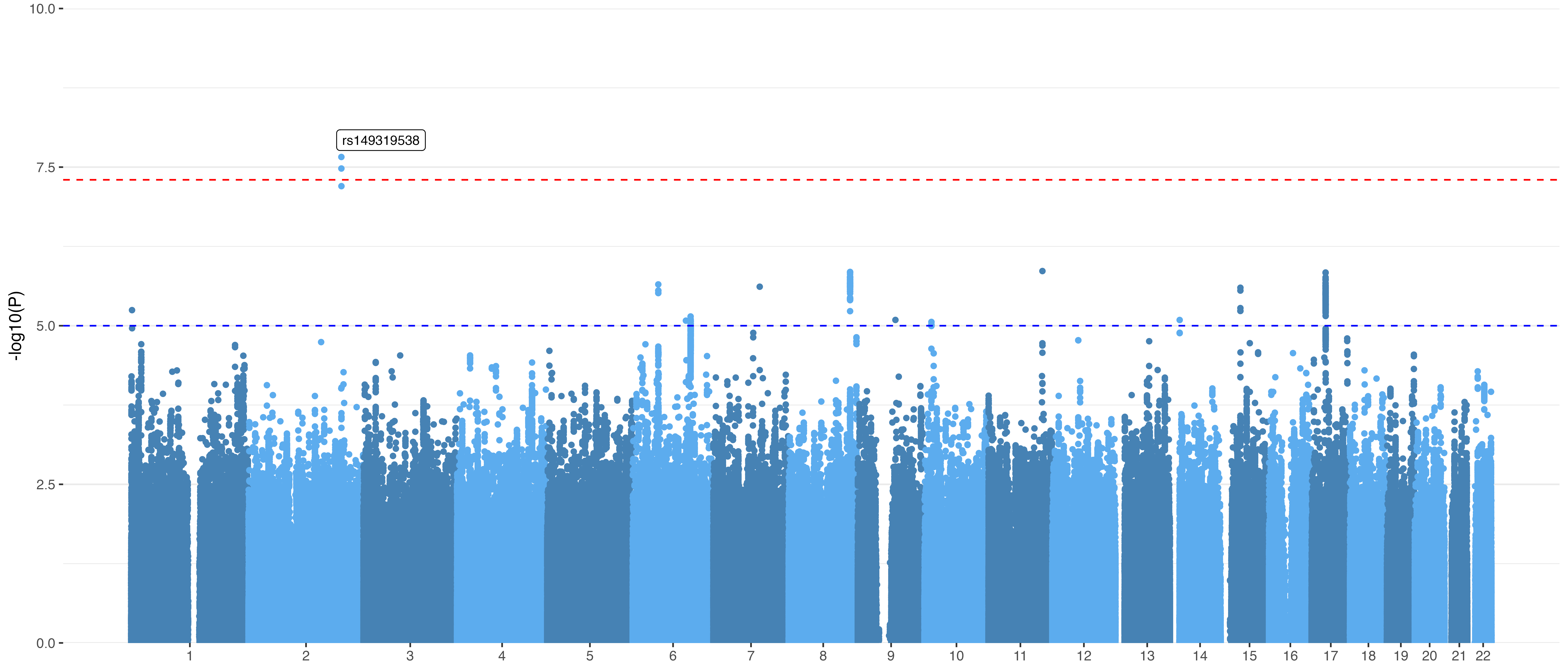


a)


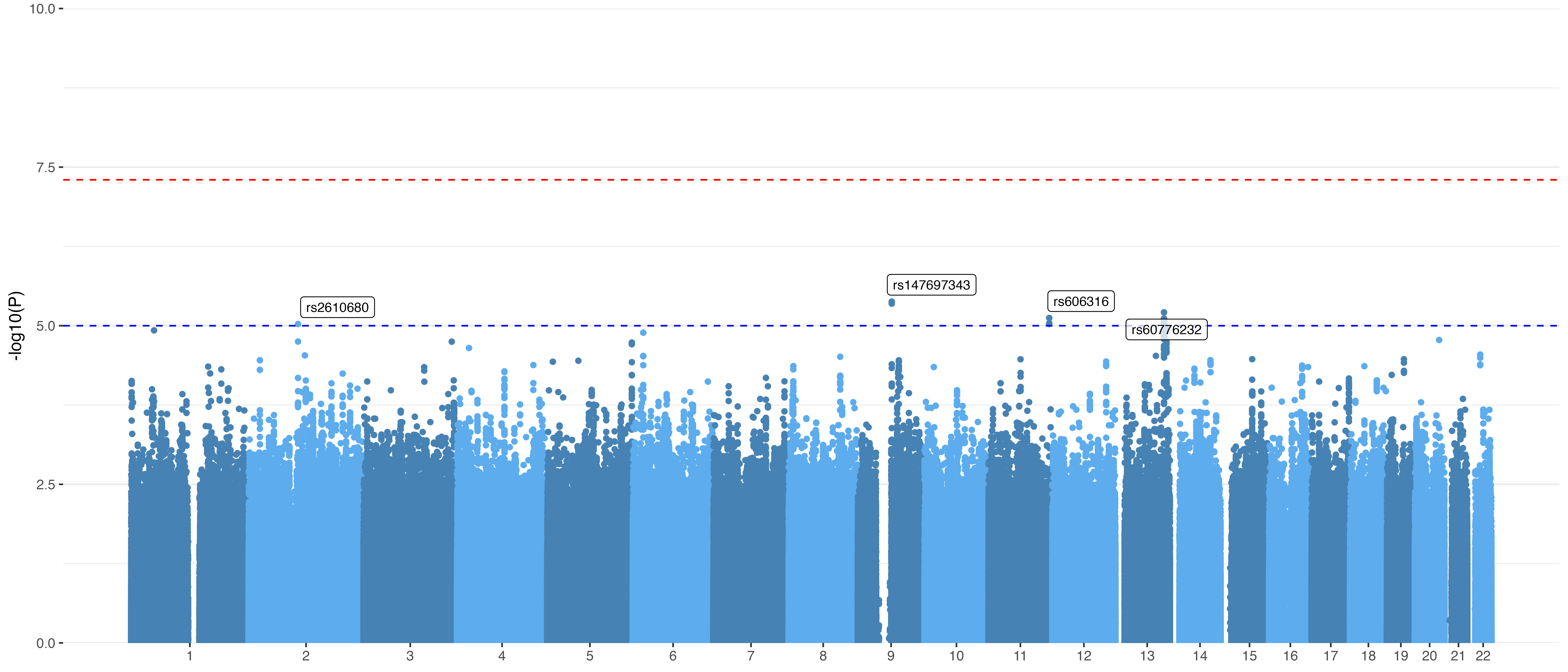


b)

## **Supplementary Figure 4. Manhattan plots of the Million Veteran Program genome-wide association studies.** Panel A shows the results among European-like ancestry individuals, and Panel B shows the results among African-like ancestry individuals.


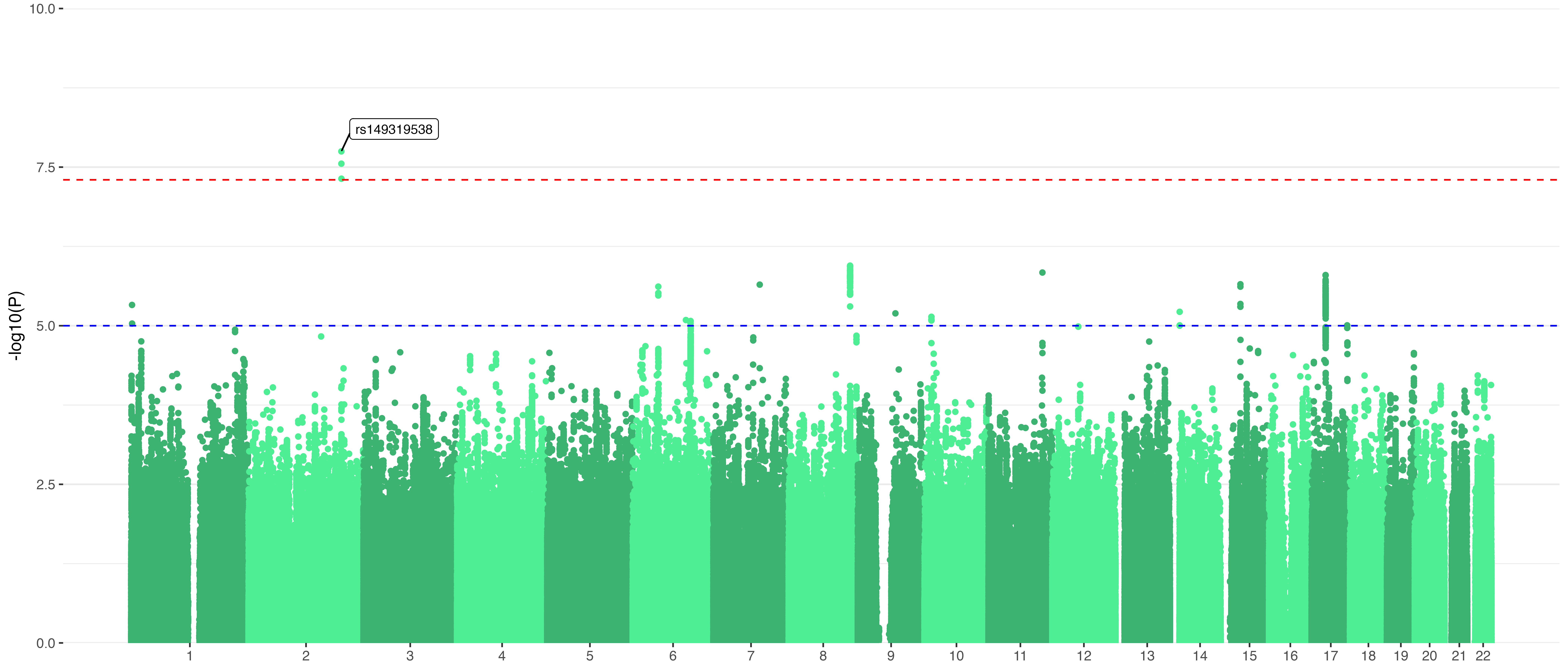


a)


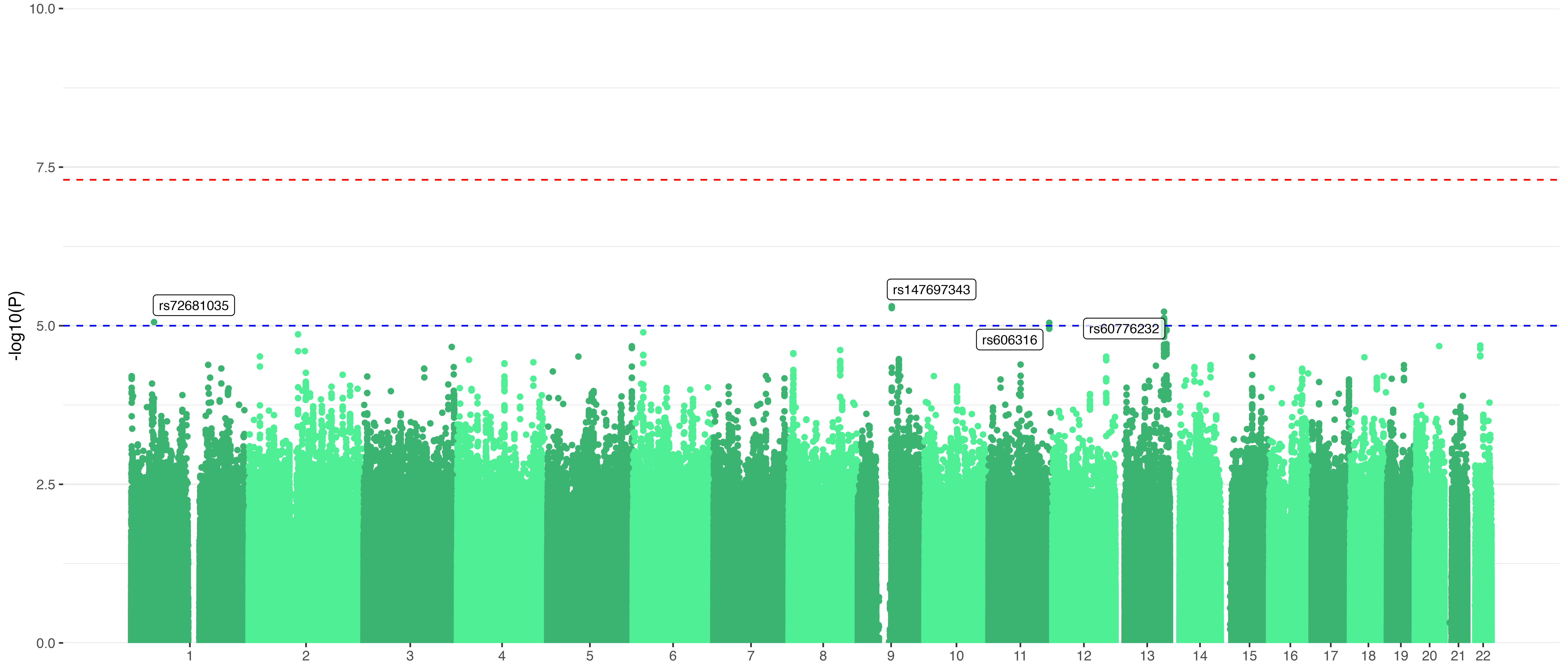


b)

## **Supplementary Figure 5. Manhattan plots of the Million Veteran Program genome-wide association studies controlling for maximum daily dosage.** Panel A shows the results among European-like ancestry individuals, and Panel B shows the results among African-like ancestry individuals.


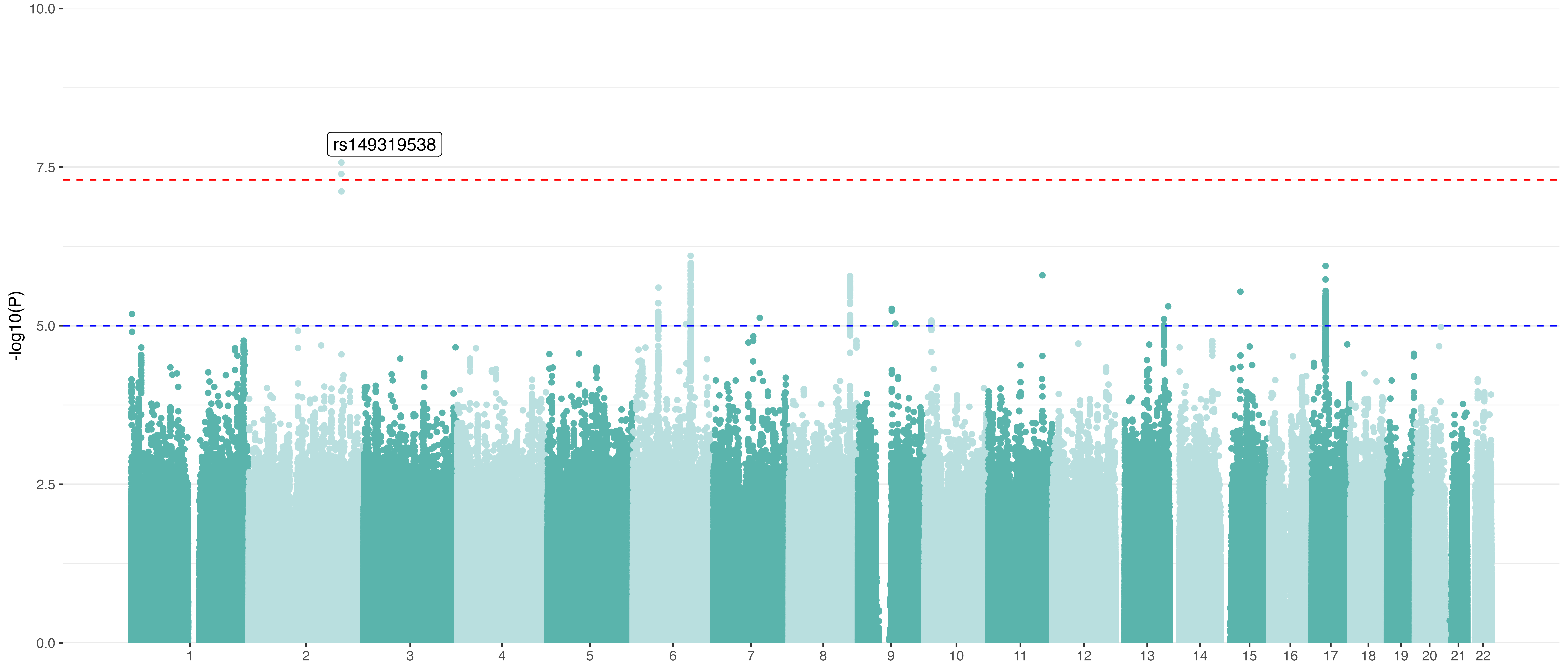


## **Supplementary Figure 6. Manhattan plot of the cross-ancestry Million Veteran Program genome-wide association meta-analysis.** The lead single-nucleotide polymorphism is annotated.


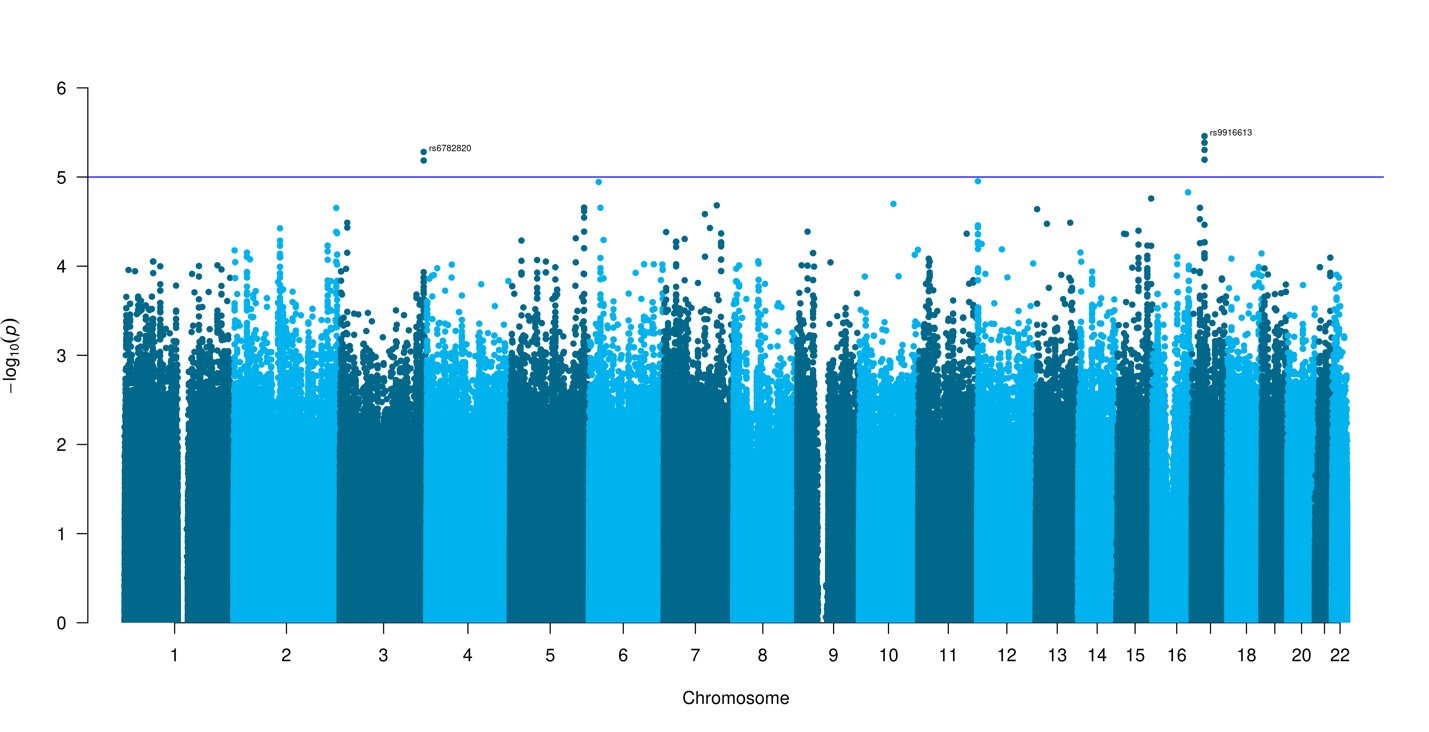

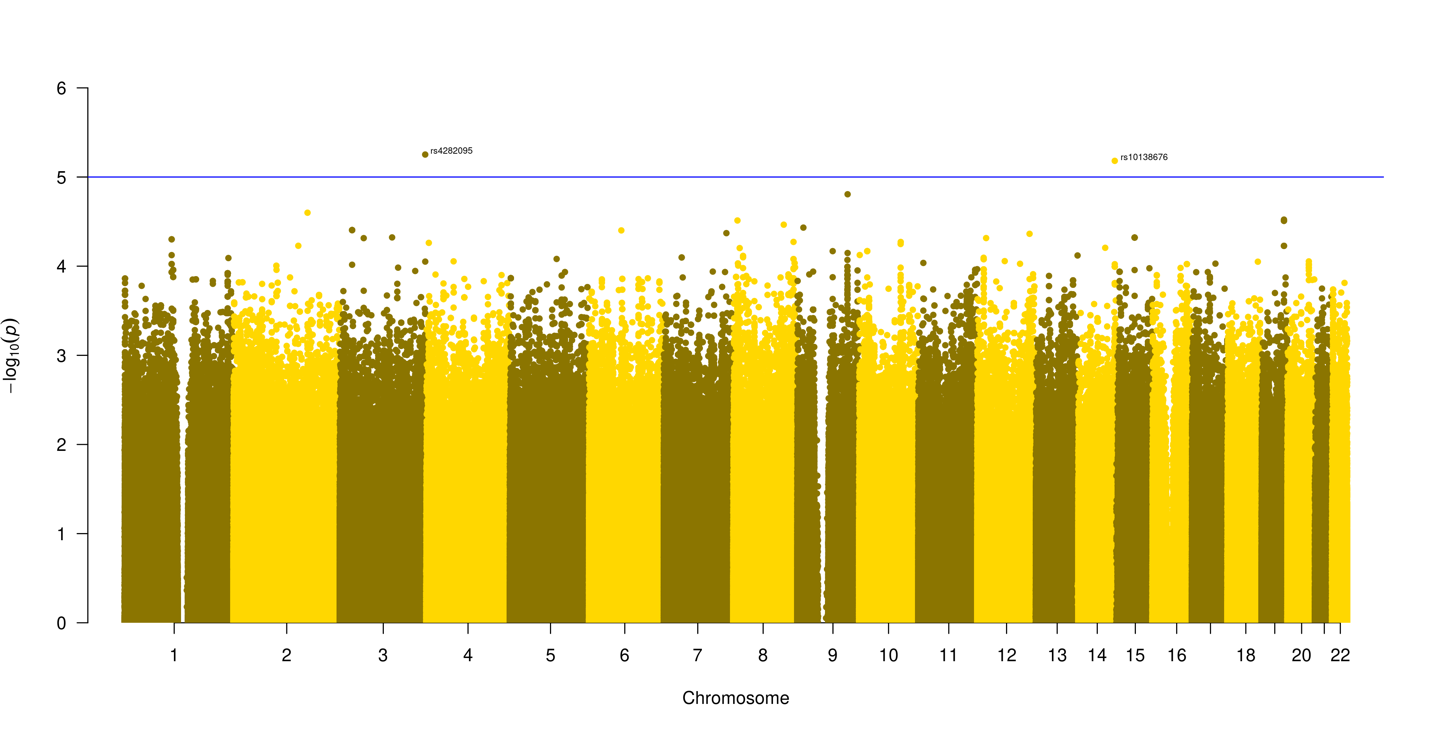


a)

b)

## **Supplementary Figure 7. Manhattan plots of the Indivior genome-wide association studies.** Panel A shows the results among European-like ancestry individuals, and Panel B shows the results among African-like ancestry individuals. The blue line indicates the genome-wide suggestive threshold at 1*10^-05^; Lead single-nucleotide polymorphisms at this threshold are annotated by rsID.


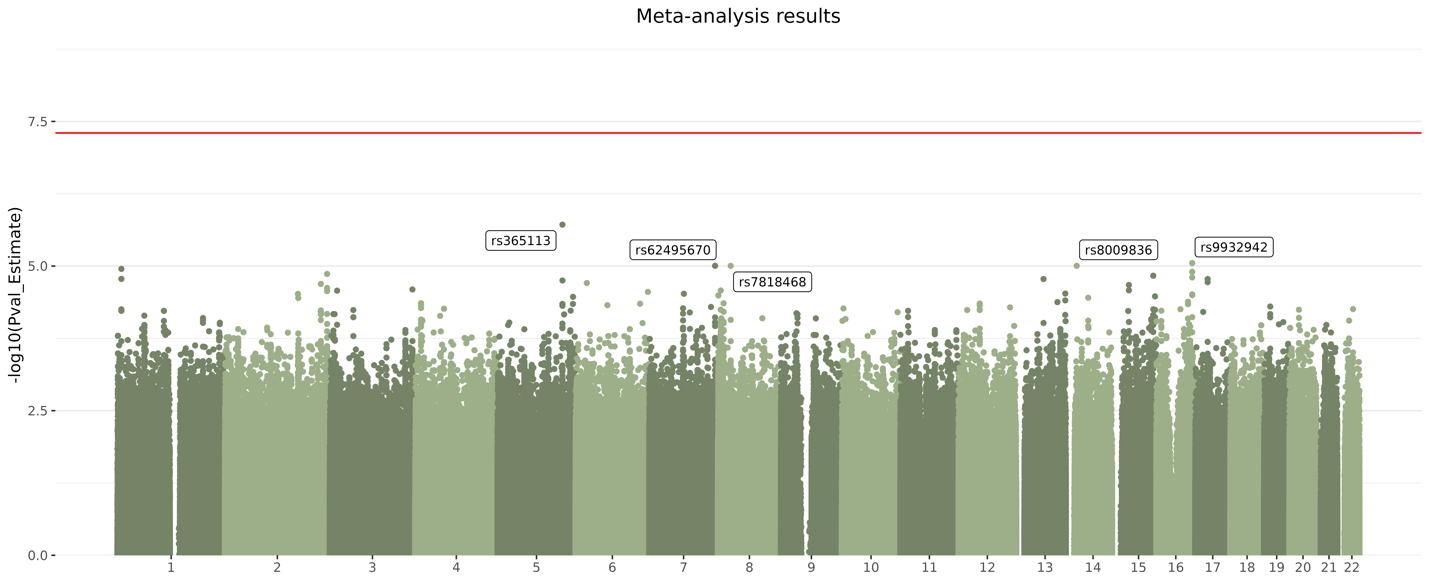


## **Supplementary Figure 8. Manhattan plot of the cross-ancestry Indivior genome-wide association meta-analysis.** The blue line indicates the genome-wide suggestive threshold at 1*10^-05^; Lead single-nucleotide polymorphisms at this threshold are annotated by rsID.


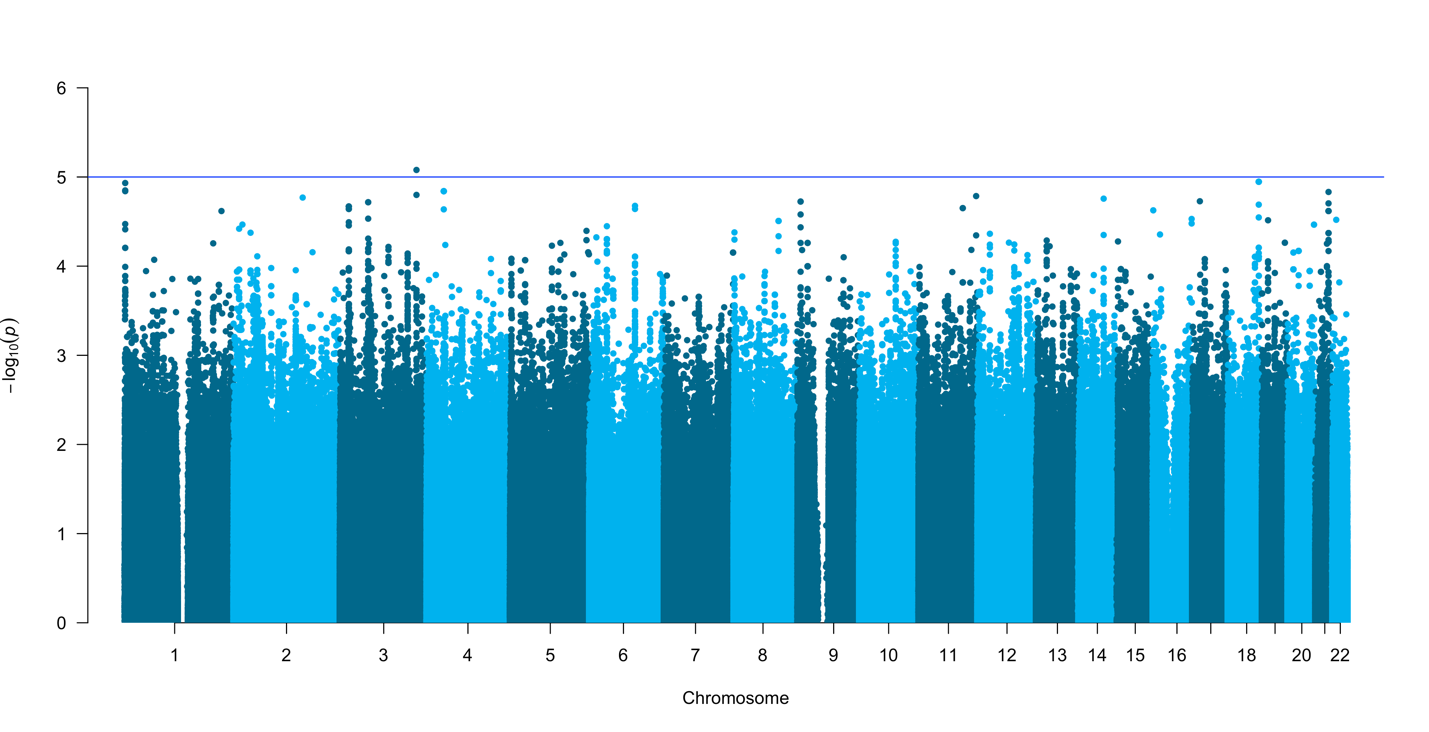


b)

a)


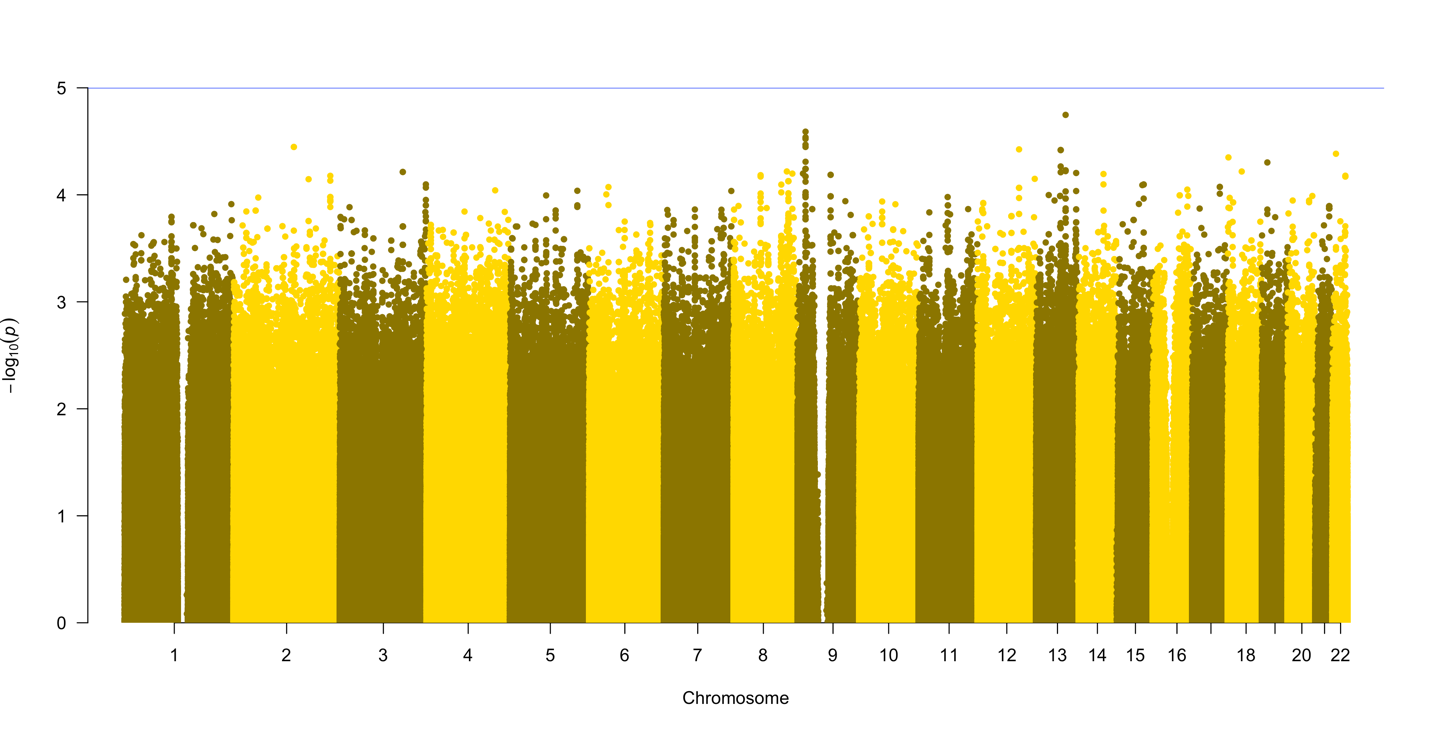


## **Supplementary Figure 9. Manhattan plots of the Indivior genome-wide association sensitivity analyses.** Analysis was performed using a definition of treatment response in the Indivior sample such that only individuals with consistently negative UDS throughout the treatment period are considered responders. Panel A shows the results among European-like ancestry individuals, and Panel B shows the results among African-like ancestry individuals. The blue line indicates the genome-wide suggestive threshold at 1*10^-05^.


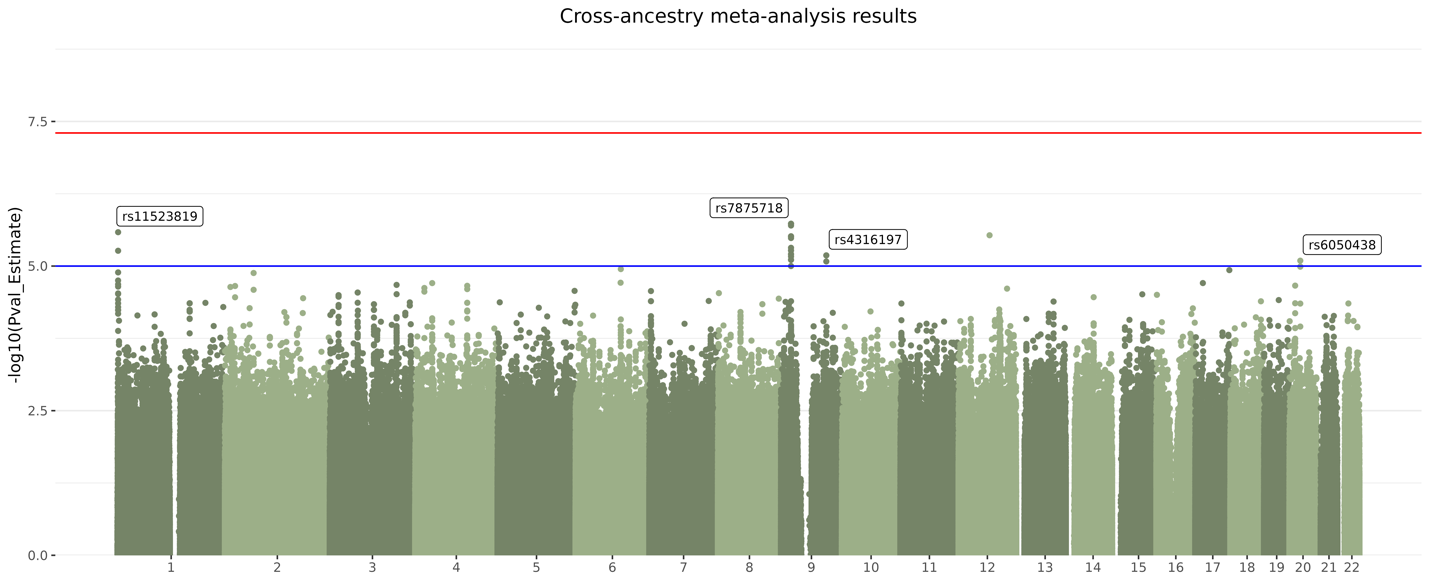


## **Supplementary Figure 10. Manhattan plot of the cross-ancestry Indivior genome-wide association sensitivity meta-analysis.** Analysis was performed using a definition of treatment response in the Indivior sample such that only individuals with consistently negative UDS throughout the treatment period are considered responders. Panel A shows the results among European-like ancestry individuals, and Panel B shows the results among African-like ancestry individuals. The blue line indicates the genome-wide suggestive threshold at 1*10^-05^; Lead single nucleotide polymorphisms at this threshold are annotated by rsID.


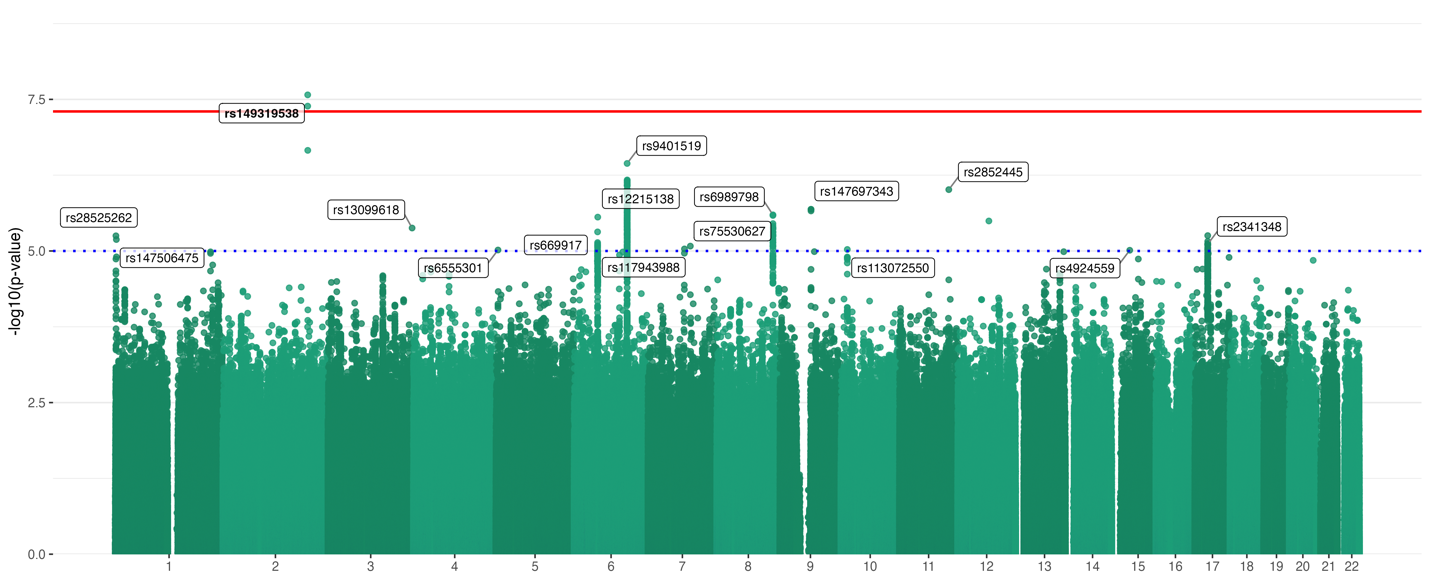


## **Supplementary Figure 11. Manhattan plot of the cross-ancestry, cross-cohort genome-wide association sensitivity meta-analysis.** Analysis was performed using a definition of treatment response in the Indivior sample such that only individuals with consistently negative UDS throughout the treatment period are considered responders. Panel A shows the results among European-like ancestry individuals, and Panel B shows the results among African-like ancestry individuals. The blue line indicates the genome-wide suggestive threshold at 1*10^-05^; Lead single nucleotide polymorphisms at this threshold are annotated by rsID.


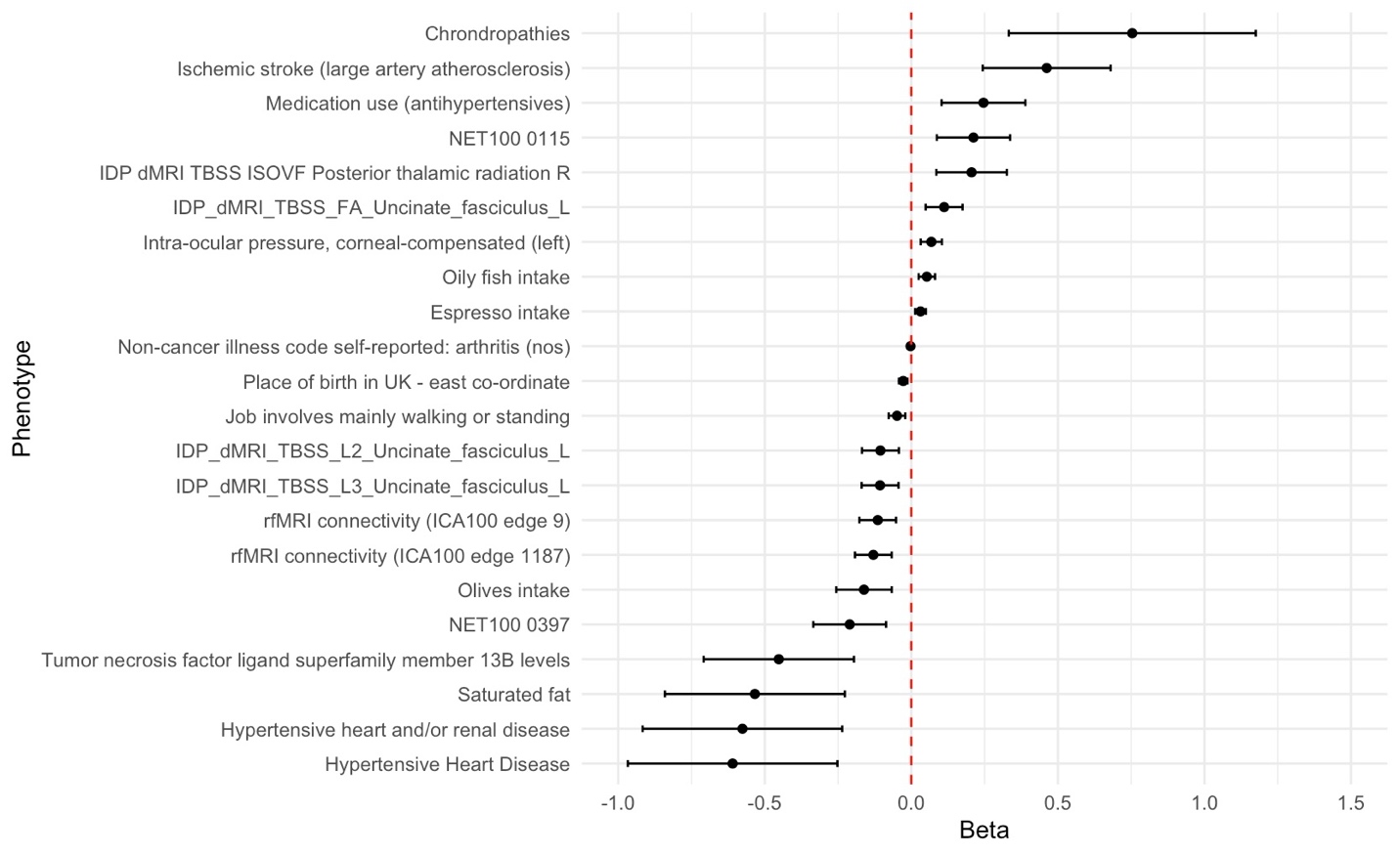


## **Supplementary Figure 12. PheWAS results for the lead single-nucleotide polymorphism.**

PheWAS was performed using the *ieugwasr* package.


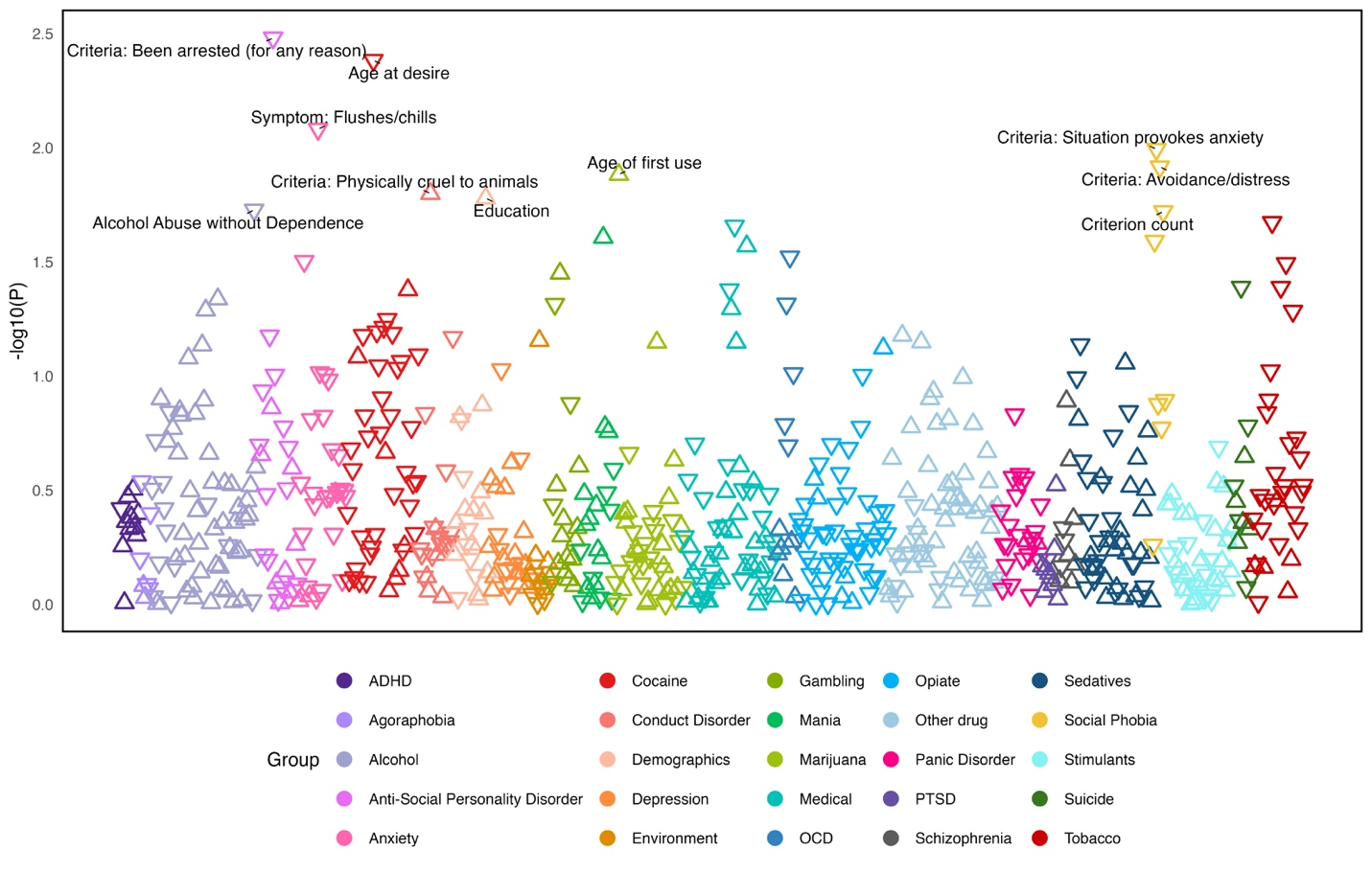


## **Supplementary Figure 13. PheWAS of PGS in Yale-Penn within EUR individuals.**

Y-axis represents log-transformed uncorrected p-values, and position of arrow represents direction of association (up = positive, down = negative).


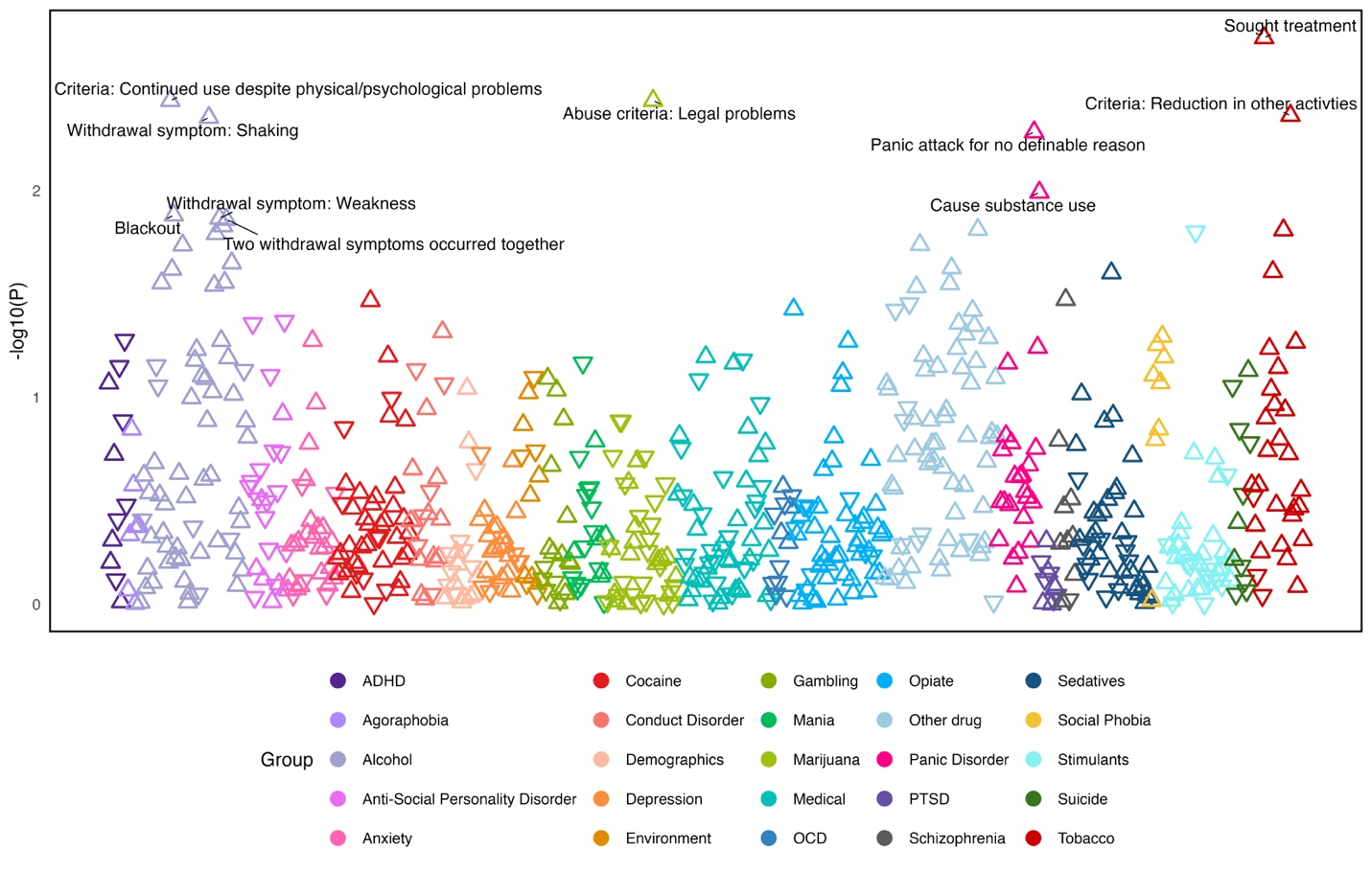


## **Supplementary Figure 14. PheWAS of PGS in Yale-Penn within AFR individuals.**

Y-axis represents log-transformed uncorrected p-values, and position of arrow represents direction of association (up = positive, down = negative).

**VA Million Veteran Program:**

**Core Acknowledgements for Publications**

**March 2024**

**MVP Program Office**

- Sumitra Muralidhar, Ph.D., Program Director

US Department of Veterans Affairs, 810 Vermont Avenue NW, Washington, DC 20420

- Jennifer Moser, Ph.D., Associate Director, Scientific Programs

US Department of Veterans Affairs, 810 Vermont Avenue NW, Washington, DC 20420

- Jennifer E. Deen, B.S., Associate Director, Cohort & Public Relations

US Department of Veterans Affairs, 810 Vermont Avenue NW, Washington, DC 20420

**MVP Executive Committee**

- Co-Chair: Philip S. Tsao, Ph.D.

VA Palo Alto Health Care System, 3801 Miranda Avenue, Palo Alto, CA 94304

- Co-Chair: Sumitra Muralidhar, Ph.D.

US Department of Veterans Affairs, 810 Vermont Avenue NW, Washington, DC 20420

- J. Michael Gaziano, M.D., M.P.H.

VA Boston Healthcare System, 150 S. Huntington Avenue, Boston, MA 02130

- Elizabeth Hauser, Ph.D.

Durham VA Medical Center, 508 Fulton Street, Durham, NC 27705

- Amy Kilbourne, Ph.D., M.P.H.

VA HSR&D, 2215 Fuller Road, Ann Arbor, MI 48105

- Michael Matheny, M.D., M.S., M.P.H.

VA Tennessee Valley Healthcare System, 1310 24^th^ Ave. South, Nashville, TN 37212

- Dave Oslin, M.D.

Philadelphia VA Medical Center, 3900 Woodland Avenue, Philadelphia, PA 19104

**MVP Co-Principal Investigators**

- J. Michael Gaziano, M.D., M.P.H.

VA Boston Healthcare System, 150 S. Huntington Avenue, Boston, MA 02130

- Philip S. Tsao, Ph.D.

VA Palo Alto Health Care System, 3801 Miranda Avenue, Palo Alto, CA 94304

**MVP Core Operations**

- Jessica V. Brewer, M.P.H., Director, MVP Cohort Operations

VA Boston Healthcare System, 150 S. Huntington Avenue, Boston, MA 02130

- Mary T. Brophy M.D., M.P.H., Director, VA Central Biorepository

VA Boston Healthcare System, 150 S. Huntington Avenue, Boston, MA 02130

- Kelly Cho, M.P.H, Ph.D., Director, MVP Phenomics

VA Boston Healthcare System, 150 S. Huntington Avenue, Boston, MA 02130

- Lori Churby, B.S., Director, MVP Regulatory Affairs

VA Palo Alto Health Care System, 3801 Miranda Avenue, Palo Alto, CA 94304

- Scott L. DuVall, Ph.D., Director, VA Informatics and Computing Infrastructure (VINCI)

VA Salt Lake City Health Care System, 500 Foothill Drive, Salt Lake City, UT 84148

- Saiju Pyarajan Ph.D., Director, Data and Computational Sciences

VA Boston Healthcare System, 150 S. Huntington Avenue, Boston, MA 02130

- Robert Ringer, Pharm.D., Director, VA Albuquerque Central Biorepository

New Mexico VA Health Care System, 1501 San Pedro Drive SE, Albuquerque, NM 87108

- Luis E. Selva, Ph.D., Director, MVP Biorepository Coordination

VA Boston Healthcare System, 150 S. Huntington Avenue, Boston, MA 02130

- Shahpoor (Alex) Shayan, M.S., Director, MVP PRE Informatics

VA Boston Healthcare System, 150 S. Huntington Avenue, Boston, MA 02130

- Brady Stephens, M.S., Principal Investigator, MVP Information Center

Canandaigua VA Medical Center, 400 Fort Hill Avenue, Canandaigua, NY 14424

- Stacey B. Whitbourne, Ph.D., Director, MVP Cohort Development and Management

VA Boston Healthcare System, 150 S. Huntington Avenue, Boston, MA 02130

**MVP Publications and Presentations Committee**

- Co-Chair: Themistocles L. Assimes, M.D., Ph. D

VA Palo Alto Health Care System, 3801 Miranda Avenue, Palo Alto, CA 94304

- Co-Chair: Adriana Hung, M.D.; M.P.H

VA Tennessee Valley Healthcare System, 1310 24^th^ Ave. South, Nashville, TN 37212

- Co-Chair: Henry Kranzler, M.D.

Philadelphia VA Medical Center, 3900 Woodland Avenue, Philadelphia, PA 19104
